# Can CT Virtual Cystoscopy Replace Conventional Cystoscopy in Early Detection of Bladder Cancer?

**DOI:** 10.1155/2015/926590

**Published:** 2015-10-27

**Authors:** Sachin Abrol, Ankush Jairath, Sanika Ganpule, Arvind Ganpule, Shashikant Mishra, Ravindra Sabnis, Mahesh Desai

**Affiliations:** Muljibhai Patel Urological Hospital, Dr. Varendra Desai Road, Nadiad, Gujarat 387001, India

## Abstract

*Aim*. To correlate findings of conventional cystoscopy with CT virtual cystoscopy (CTVC) in detecting bladder tumors and to evaluate accuracy of virtual cystoscopy in early detection of bladder cancer. *Material and Method*. From June 2013 to June 2014, 50 patients (46 males, four females) with history and investigations suggestive of urothelial cancer, with mean age 62.76 ± 10.45 years, underwent CTVC by a radiologist as per protocol and subsequently underwent conventional cystoscopy (CPE) the same day or the next day. One urologist and one radiologist, blinded to the findings of conventional cystoscopy, independently interpreted the images, and any discrepant readings were resolved with consensus. *Result*. CTVC detected 23 out of 25 patients with bladder tumor(s) correctly. Two patients were falsely detected as negative while two were falsely labeled as positive in CTVC. Virtual and conventional cystoscopy were comparable in detection of tumor growth in urinary bladder. The sensitivity, specificity, positive predictive value, and negative predictive value of virtual cystoscopy were 92% each. *Conclusion*. CTVC correlates closely with the findings of conventional cystoscopy. Bladder should be adequately distended and devoid of urine at the time of procedure. However, more studies are required to define the role of virtual cystoscopy in routine clinical practice.

## 1. Introduction

The most common cancer of the lower urinary tract is bladder tumor, with recurrence as one of its main troublesome features associated depending upon different stages and grades [1, 2]. Therefore, close monitoring of the patients is required at regular intervals. The mainstay of diagnosis and follow-up of bladder neoplasia as of now is conventional cystoscopy [1] but it is an invasive procedure. Replacing invasive diagnostic procedures with noninvasive sensitive and specific imaging techniques is a growing trend in medicine today.

With computer assisted rapid image acquisition and three-dimensional image reconstruction by commercially available software, virtual reality imaging has been developed. This can be applied to many organs including the colon, stomach, bronchus, and bladder [3–8]. Because of its simple luminal morphology, relatively small volume, and absence of involuntary peristalsis, urinary bladder may possibly be an ideal intra-abdominal organ for virtual endoscopy [9].

The accuracy of detecting bladder lesions (<1 cm) by computed tomography virtual cystoscopy (CTVC) has been variously reported ranging within 60–100% [7, 10–12]. Even bladder lesions <5 mm have also been reportedly detected by some authors utilizing CTVC [13–15], while others have found the visualization of such lesions to be difficult [10]. This technology is considered safe for bladder cancer follow-up with detection rates comparable to conventional cystoscopy.

Conventional cystoscopy (CPE) on the other hand is invasive and time consuming and, especially in males, the evaluation of bladder neck and narrow neck diverticular tumors is limited. In addition, there is risk of urinary sepsis and urethral injury, which can lead to late urethral strictures [5] so virtual cystoscopy can play increasing role as a first-line screening test to evaluate patients at risk of bladder cancer or who present with symptoms like hematuria in near future.

In this study we report our experience with CTVC in detecting bladder tumors in terms of sensitivity, specificity, positive predictive value, and negative predictive value.

## 2. Patients and Methods

### 2.1. Patients

After getting informed consent and obtaining ethics committee approval, all patients with history and investigations suggestive of urothelial cancer who serially presented in outpatient department from June 2013 to June 2014 were included in the study. The age ranged from 30 to 83 years (mean age 62.76 ± 10.45 years), 46 were males, and four were females. A focused history examination, urine analysis/culture/cytology for malignant cells, renal function tests, and ultrasonography of kidney, ureters, and bladder (KUB) region, was performed in all the patients. Out of the 50 patients, 21 patients had presented to our outpatient department first time with gross painless hematuria and symptoms suggestive of bladder tumor and the remaining 29 patients were already proven cases of bladder tumor that had undergone TURBT and were admitted for follow-up check cystoscopy.

### 2.2. Technique

Virtual cystoscopy began drainage of residual urine by placing a 12 fr Foleys catheter into the bladder. It was then insufflated with 300–400 mL of room air using 60 cc syringe and clamp, according to the bladder capacity and tolerance. In few cases, the catheter was withdrawn to the distal penile urethra so that the urethra may also be visualized at the time of virtual cystoscopy. Initially, a scout view was obtained to locate the bladder and confirm its adequate distention with the patient in the supine position. Subsequently, single breath hold CT was performed with Multidetector CT (Bright speed, GE Health care) with 1 mm collimation, 120 KV, 250 mA, and 7–10 mm/sec table speed. Images were reconstructed at 1.25 mm intervals by using the minimal field of view measured from the inner aspect of the middle of the pelvis. The procedure was repeated with the patient in prone position using the same CT parameters. The data was then analyzed using software for interactive intraluminal navigation with a volume rendering algorithm. One urologist and one radiologist, blinded to the findings of conventional cystoscopy, independently interpreted the images, and any discrepant readings were resolved with consensus. Using the multiplanar reformation from the source images, the central observation point was defined in the middle of the bladder. The camera of the virtual cystoscopy was placed in the center of the bladder and thereafter advanced to each quadrant and the findings were then evaluated from various angles.

The number, size, location, and morphological features of the lesions were evaluated and virtual images were obtained with the patients in both the prone and the supine positions. Each lesion was then classified as polypoidal lesion, a sessile mass, or wall thickening. A discrete lesion was considered polypoidal if it was taller than it was wide, while a sessile mass was defined as a lesion that was wider at the base. A lesion was characterized as wall thickening when there was elevation of the bladder wall without a discrete mass.

Subsequently, each of the patients underwent conventional cystoscopy (CPE) the same day or the next day. The urologists, who were not preminded with virtual cystoscopic findings, performed cystoscopic examinations using rigid wide angle telescopes. They were instructed to determine the number, location, and morphology of the bladder lesions by drawing or video-recording.

CPE findings were used as reference standard to evaluate the sensitivity, specificity, positive predictive value, and negative predictive value of CTVC for detection of bladder tumors.

## 3. Result

Out of 50 patients included in the study, twenty-one (21) patients were evaluated for gross painless hematuria and the remaining twenty-nine (29) patients were already proven cases of bladder tumor who had undergone TURBT in our institution previously and were on follow-up check cystoscopy. Out of 21 patients evaluated for hematuria, transitional cell carcinoma, benign prostate hyperplasia, and cystitis were diagnosed in 17, 2, and 2 patients, respectively, on CPE. New growths were detected in 8 out of 29 patients undergoing check CPE.

Both CTVC and CPE were well tolerated by all the patients without any complications. Images in 45 out of 50 virtual cystoscopies were of excellent or good quality, with adequate bladder distention and minimum residual urine. Images in 5 patients were suboptimal due to inadequate bladder distention and moderate residual urine.

CTVC detected 23 out of 25 patients with bladder tumor(s) correctly (Table 1(a)). Out of two patients who were falsely detected as negative on virtual cystoscopy, one patient had a sessile tumor measuring around 1.0 cm on right lateral wall on CPE and CTVC diagnosed it as normal while another patient with 1 cm tumor near bladder neck was not picked on CTVC probably due to residual urine. Two patients were falsely labeled as positive in CTVC. In one patient, two lesions of 0.3 mm were suspected on the right lateral wall, near the previous site of TURBT, but no lesion was found on CPE. This was due to inadequate bladder distention and bladder trabeculations present in this patient which led to the misinterpretation of findings. In another patient who was labeled as bladder tumor at bladder neck by CTVC, only prostatomegaly was detected on CPE.

A total of 80 tumor lesions were detected in 25 positive patients in CPE. Single tumor was found in thirteen patients, two lesions were detected in two patients, and more than two were found in ten patients. Other than one CIS and four other tumors which were missed on CTVC, all other tumor lesions were identified with accuracy with regard to position and distribution as on CPE.

Morphologically, identified tumors were categorized into three types, papillary in 60 lesions, sessile in nineteen, and CIS in one. In the present study, virtual and conventional cystoscopy were comparable in detection of tumor growth in urinary bladder. The tumor size ranged from 0.2 cm to 5.0 cm in maximum dimension.

**Figure 1:**
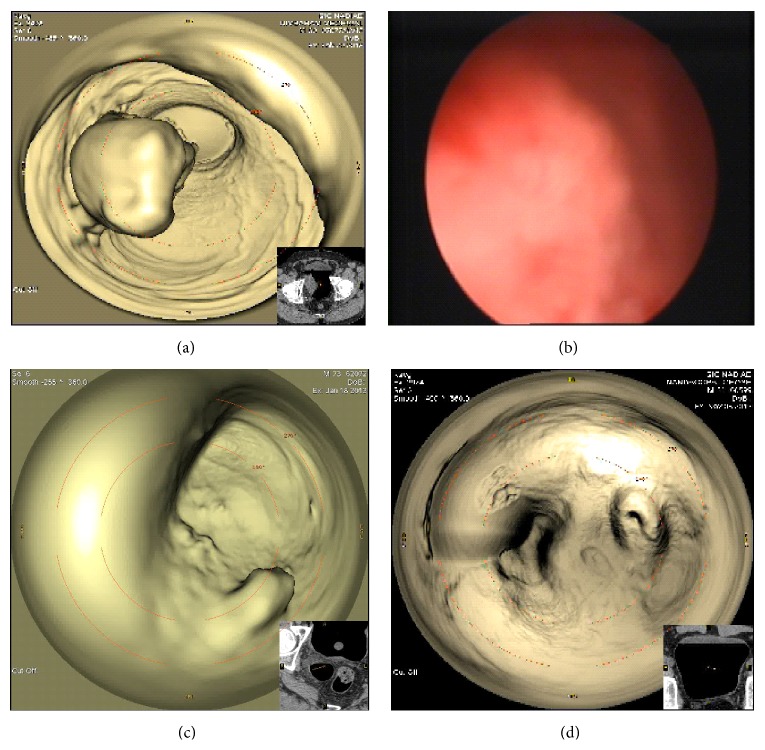
(a-b) Large right lateral wall growth in a patient presenting with gross painless hematuria on CTVC and CPE, respectively. (c) CTVC image of inside the bladder diverticulum showing papillary lesions. (d) Follow-up case of TCC bladder with normal findings on CTVC.

Among the visualized 80 tumors, 31 were located at the right lateral wall (Figures 1(a)-1(b)), sixteen on left lateral wall, twelve at the posterior wall, ten at the base, six at the dome, and four lesions in the diverticulum (Figure 1(c)) and one was a case of CIS.

Other results were shown in Tables 1(b), 2(a)-2(b), and 3.

## 4. Discussion

Bladder cancer is one of the most common urological malignancies, with the need for long-term follow-up. Because bladder tumors have a tendency towards multifocality and recurrence, it seems very important to find out diagnostic techniques that are less invasive and at the same time highly sensitive.

Although several imaging techniques like intravenous urography, ultrasound, CT, and magnetic resonance imaging have been used for detecting bladder tumor, none of them may be completely sensitive in all aspects. While overall CT may be a useful radiological tool, its sensitivity appears to be low particularly for detection of small bladder lesions. More negative findings on CT may warrant further evaluation with conventional CPE [3, 4, 16].

Conventional CPE has traditionally served as the reference standard for detecting intravesical lesions [3, 5]. However, CPE has some limitations. The evaluation of bladder neck, anterior wall, and diverticulum (narrow neck) is difficult. Primary intradiverticular carcinomas are rare but diagnosis is often difficult with conventional method [6, 17–19]. Marked hematuria is another factor that limits the technical success of cystoscopy, thereby decreasing its reliability. Cystoscopy is performed under general or local anesthesia and it is an invasive and uncomfortable procedure for the patient and complications like infection, bladder perforation, scarring, and stricture of urethra have been observed [3, 6].

Recently introduced virtual endoscopy seems to be an advantageous tool to detect and evaluate bladder lesions. Virtual endoscopy has been most widely applied to the imaging of colon and many investigators report its feasibility in the detection of colorectal polyps [20, 21]. After the first report of virtual cystoscopy in study by Vining et al., there have been a lot of studies on the utility of virtual cystoscopy of bladder. Urinary bladder is a good organ for virtual cystoscopy because of its simple luminal morphology, relatively small volume, and absence of involuntary peristalsis. Therefore, virtual cystoscopic rendering of bladder takes less time to navigate and does not require great skill on the part of operator [4, 5, 22]. According to a study by Kim et al., virtual cystoscopy was found superior to multiplanar reconstruction and source CT images for lesion detection in contrast material filled bladder [21].

A recent meta-analysis of 26 studies done by Qu et al. has reported pooled sensitivity and specificity of virtual cystoscopy to be 93.9% and 98.1%, respectively [23]. CTVC is a relatively noninvasive emerging tool in the diagnostic armamentarium of bladder pathology.

CTVC in our study was obtained by combined use of supine and prone images, as reported by others [9, 16, 24, 25]. We calculated the data patientwise as well as lesionwise. Both sensitivity and specificity in our study turned out to be 92%. As the multiplicity of the bladder tumors may change the treatment plan, we analyzed sensitivity and specificity of CTVC in detecting number of tumors (Table 2(a)). While sensitivity (94.9%) increases, specificity decreases to 83.3% (Table 2(b)).

Three-dimensional images generated from volumetric data obtained from helical CT imaging were used in our study. Since the work published by Vining et al. [4], there have been several studies that discussed the utility of VC in bladder lesions [5, 6, 22, 26, 27]. To date, two techniques that use either air or contrast material to fill bladder have been used for VC [4, 5, 26, 27]. The results obtained from intravenous contrast media were adequately similar to those obtained with air contrast. However, presence of faint artifacts within obtained images when using IV contrast makes the images obtained from air contrast clearer and sharper.

In our study, we found that virtual cystoscopy is a feasible technique for use in detection of bladder lesions greater than 3 mm. Narumi et al. [27] identified 77% of lesions smaller than 10 mm. Fenlon et al. [25] identified all the lesions smaller than 10 mm in their study of 13 patients. However, these studies retrospectively evaluated bladder lesions that had been confirmed on CPE.

Though all patients tolerated the CTVC procedure well, still our study lacked any objective data with respect to tolerance of the procedure in form of pain scores or validated questionnaire. This could be included as an objective in future multicenter trial. Also, we did not record the exact timing to complete the CTVC procedure (including patient preparation, instillation of air, CT imaging, processing of the acquired data, and analysis) which forms one of the limitations of our study. Further randomized studies will be needed to validate and define the role of CTVC in routine practice.

## 5. Conclusion

1. CT virtual cystoscopy correlates closely with the findings of conventional cystoscopy in our study.
2. The sensitivity, specificity, positive predictive value, and negative predictive value of virtual cystoscopy were 92% each, in our study.

The salient procedure points noted in our study are as follows:

- The bladder should be adequately distended at the time of procedure; otherwise, crumpling of wall may give artefacts in the study.
- The bladder should be devoid of urine at the time of examination. Virtual cystoscopy should be done both in supine and in prone positions.
- In grossly trabeculated bladder, small papillary growth may be overlooked, so close inspection is required.
- The dictum followed should be a systematic inspection of bladder starting by keeping centre of bladder as the region of interest and moving systematically from normal to abnormal area/surface.

## Figures and Tables

**(a):** 

Parameter	Positive result (tumor detected) (*N*)	Negative result (tumor not detected) (*N*)	True positive (*N*)	True negative (*N*)	False positive (*N*)	False negative (*N*)
CPE^*∗*^	25	25	25	25	0	0
CTVC^*∗∗*^	25	25	23	23	2	2

^*∗*^CPE: cystopanendoscopic examination was taken as reference standard, *N*: number of patients, and ^*∗∗*^CTVC: computed tomography virtual cystoscopy.

**(b):** 

Parameter	Sensitivity	Specificity	Ppv	Npv
CTVC	92%	92%	92%	92%

Ppv: positive predictive value and Npv: negative predictive value.

**(a):** 

Parameter	CTVC positive	CTVC negative
CPE^*∗*^ positive	75	5
CPE negative	4	25

^*∗*^CPE: cystopanendoscopic examination was taken as reference standard and CTVC: computed tomography virtual cystoscopy.

**(b):** 

Parameter	Sensitivity	Specificity	Ppv	Npv
CTVC	94.90%	83.30%	93.75	86.21

Ppv: positive predictive value and Npv: negative predictive value.

**Table 3:** Number of bladder growths detected by virtual and conventional cystoscopies according to size.

Size of tumor	≥1 cm	0.3–1.0 cm	<0.3 cm
CPE (*n* = 80)	19	58	2 + CIS
CTVC (*n* = 75)	19	56	0

CIS: carcinoma in situ.
